# Complete genome sequence of strain AEG42_29 (DSM 112329), a member of the genus *Paraconexibacter* isolated from soil

**DOI:** 10.1128/mra.01163-24

**Published:** 2024-12-16

**Authors:** Selma Vieira, Katharina Huber, Alicia Geppert, Jörg Overmann

**Affiliations:** 1 Leibniz Institute DSMZ-German Collection of Microorganisms and Cell Cultures, Braunschweig, Germany; 2 Technical University of Braunschweig, Braunschweig, Germany; DOE Joint Genome Institute, Berkeley, California, USA

**Keywords:** soil bacterium, complete genome, *Paraconexibacter*

## Abstract

We report the complete genome sequence of strain AEG42_29, a representative of the genus *Paraconexibacter* isolated from a temperate grassland soil. Its genome codes for several genes involved in stress response, defense, and virulence, which may help this bacterium cope with fluctuating conditions in the soil environment.

## ANNOUNCEMENT

Bacteria affiliated with the order *Solirubrobacterales* are ubiquitous and abundant in different soils (1). They are often reported to be associated with plant rhizospheres (2, 3), and some have the capacity to degrade pollutants such as ibuprofen (4). Despite their importance, they are underexplored and only a total of 22 species have been described so far within this entire order.

Strain AEG42_29 was isolated from a temperate grassland soil, sampled in May 2014 in the framework of the German Biodiversity Exploratories in the vicinity of Gomadingen (48°23′52.82″N, 9°22′33.75″E). It was isolated by direct plating of a soil suspension (10 mM HEPES, pH 7.0) on solid SSE/HD 1:10 medium (pH 7) (5) at room temperature, as previously described (6). The almost full-length 16S rRNA gene was amplified by colony PCR with a primer pair 8f (7) and 1492r (8) (sequence accession number ON980750), and was used for an initial identification using the EzBioCloud database (9). *Paraconexibacter antarticus* 02-257^T^ (10) was identified as the closest relative of strain AEG42_29 with 99.5% sequence similarity of the 16S rRNA gene. Average nucleotide identity (ANI) between both strains was 80.1%, calculated with OrthoANIu (www.ezbiocloud.net/tools/ani) (11).

Genomic DNA was extracted using the MasterPure Gram Positive DNA Purification Kit (Biosearch Technologies, Hoddesdon, UK). A library was prepared using SMRTbell Express Template Prep Kit 2.0 (Pacific Biosciences, Menlo Park, CA, USA), according to the instructions of the manufacturer. The genome of AEG42_29 was sequenced on a PacBio Sequel II (Pacific Biosciences, Menlo Park, CA, USA) and yielded 91,614 postfiltered reads with an average read length of 8,187 bp. Because the coverage was large (848×), no correction by Illumina short-read sequencing was necessary. Long-read genome assembly was performed with HGAP (version 4) included in SMRTlink (version 10), using default parameters with exception of the target genome size, which was set to 7.5 Mbp.

The genome of AEG42_29 was 5,775,662 bp long with a G+C content of 72.5%. Prokka 1.14.6 (12) was used for annotation, and 5,377 protein coding genes were predicted, as well as 3 rRNAs (organized in one operon) and 59 tRNAs.

Comparative sequence analysis of the genome of strains AEG42_29 with available genomes of representatives of the genera *Paraconexibacter*, *Baekduia*, and *Conexibacter* was performed using the Pathosystems Resource Integration Center (PATRIC) (13). Notably, the genome of strain AEG42_29 harbored the mycosporine-like amino acid (MAA) gene cluster *mysABCD*, which was not found outside the genus *Paraconexibacter*. MAAs are known to play a role in protective UV absorption in *Cyanobacteria* (14). It also contains numerous genes involved in stress response, defense, and virulence, such as alkaline shock protein 23. The presence of this gene supports the alkaline tolerance detected for this bacterium (pH ranges from 6.5 to 10). Also of note is the presence of an operon coding for the core complex of acetophenone carboxylase (EC 6.4.1.8; units αβγδ), which is involved in the degradation of acetophenone and other aromatic ketones. Overall, this bacterium seems to be resilient to multiple stressors and thereby well equipped to successfully compete in the soil environment.

## Data Availability

The genome sequence was deposited at DDBJ/ENA/GenBank under the accession number CP114014. Raw reads are available in the European Nucleotide Archive (ENA) under the accession number ERR13866265.
