# Erratum for Vieira et al., “Complete genome sequence of strain AEG42_29 (DSM 112392), a member of the genus *Paraconexibacter* isolated from soil”

**DOI:** 10.1128/mra.01465-25

**Published:** 2026-02-12

**Authors:** Selma Vieira, Katharina Huber, Alicia Geppert, Jörg Overmann

## ERRATUM

Volume 14, no. 1, e01163-24, 2025, https://doi.org/10.1128/mra.01163-24. The article title should read as given in this erratum. There was an error in the accession number.

